# Protective and risk factors of problematic smartphone use in preteens using panel study on Korean children

**DOI:** 10.3389/fpsyt.2022.981357

**Published:** 2022-08-19

**Authors:** Jungim Yun, Gyumin Han, Hyunmi Son

**Affiliations:** ^1^College of Nursing, Pusan National University, Busan, South Korea; ^2^Nursing Department, Dong-Eui Medical Center, Busan, South Korea

**Keywords:** Addictive behavior, preteen, problematic smartphone use, peer, parenting

## Abstract

**Background:**

Increased smartphone use by children and adolescents places them at higher risk of overdependence. The problematic smartphone use of preteens is rapidly increasing. The preteen period is one of considerable developmental change and the influence of problematic smartphone use should be identified by reflecting on this change and considering its social psychological factors.

**Methods:**

This study employed a secondary analysis using data from the 10th (2017) to 11th (2018) wave of the Panel Study on Korean Children. STATA/BE 17 was used to analyze the panel logit model. Among the 1,286 participants aged 9–10 from the Panel Study on Korean Children, 342 with complete responses were selected as the participants of this study.

**Results:**

The risk group for problematic smartphone use showed an increase from 126 in 2017 to 149 in 2018. Factors influencing risk of preteens' problematic smartphone use were the child's externalizing problems (*p* = 0.015) and permissive parenting behavior (*p* = 0.003). Protective factors influencing preteens' problematic smartphone use were peer communication (*p* = 0.023), parental supervision (*p* = 0.020), and authoritative parenting behavior (*p* = 0.001).

**Conclusions:**

Preteens with externalizing problems are at a higher risk for problematic smartphone use and are therefore a group to be observed with caution. It is also required to guide them to form good relationships with friends. Finally, in the problematic smartphone use of preteens, parents are both a protective factor and a risk factor. Therefore, guidance is required so that children can behave properly.

## Introduction

Smartphones are becoming a necessity in the daily lives of most individuals due to their convenience for all users. Today's adolescents are digital natives, accustomed to learning, playing, and recreation using smartphones. Despite their numerous benefits, excessive smartphone use due to lack of self-control can lead to problems in daily life, known as Problematic Smartphone Use (PSU) (1). PSU can result in physical and psychological problems among adolescents including sleep disorders, difficulties in academic achievement and maladjustment to school life (2–5). PSU also causes a deterioration of empathy and emotional intelligence as well as interpersonal dysfunction in adolescents (6), inducing aggression that can result in behaviors such as deviance and delinquency (7, 8).

PSU in the preteen period, which corresponds to the onset of adolescence, shows the steepest increase compared to other adolescent age groups. Preteen is the transitional period of development from childhood to adolescence between the age of 9 and 12 (9) in which the characteristics of children and adolescents coexist. PSU among preteens increased by about 19% in 2020 compared to 2018, higher than the average increase of 13% among high school students (10). During the preteen period, most children own a smartphone for the first time, and as personal time without parental control increases, they actively use their smartphones for various purposes (11). Preteens with a high risk of engaging in PSU due to immature self-control are highly likely to persist with PSU in adolescence and adulthood (12).

Children's behaviors are not only affected by individual thoughts and concepts, but also by the interaction of various environmental factors such as interpersonal, community, and policy factors surrounding the child (13). Therefore, an ecological approach is required to identify the factors affecting children's problem behavior. Studies on adolescents have mainly considered individual factors such as depression, interpersonal sensitivity, school maladjustment (14, 15), and interpersonal factors such as peer relationship (16), and peer support (17). Studies focused on childhood have examined interpersonal factors such as parental smartphone use and parenting attitudes (18). Another study examined the relationship of PSU with the school environment, which is an important community factor for children and adolescents (19). However, previous studies examining the ecological factors affecting PSU were mainly conducted on adolescents or children, therefore, studies focusing on the preteen period were insufficient. In order to understand the preteens, which is an important period in PSU, it is necessary to explore the interaction of various influencing factors focusing on the preteens period.

The preteen period marks a period of rapid change in psychological and social development. The parent-centered interpersonal relationship of childhood expands and social development changes rapidly (20). It becomes important to form a peer group and forge friendships and bonds with peers during this period (21). Most previous studies examining the factors affecting PSU are cross-sectional studies on adolescents and children, and are limited, not reflecting changing preteen characteristics. To intervene in problematic smartphone use suitable for the preteen period, identifying the influencing factors that reflect developmental changes is necessary.

The purpose of this study is to identify the protective and risk factors related to PSU in preteens based on an ecological model. Moreover, the study identifies factors that reflect preteen changes and developmental characteristics using panel data and presents effective countermeasures for PSU prevention. In the ecological model, we exclude the community and policy factors that fall under the same conditions, and focus on the individual and interpersonal relationships that are important, as established in a literature review.

## Methods

### Design

This longitudinal study is a secondary analysis of data from the Panel Study on Korean Children (PSKC) 10^th^ (2017) to 11^th^ (2018) wave investigating problematic smartphone use.

### Setting and participants

In the PSKC, children born in 2008 at medical institutions nationwide will be followed up until 2027 to track their growth and development. The PSKC uses stratified multi-step sampling and data are collected by dividing the country into six regions. The number of births per year in each region is proportionally allocated. PSKC data were acquired from a balanced panel surveyed at regular intervals. In this study; since raw data measuring PSU, which is available to the general public, is in the 10 and 11 PSKC, 9–10-years-old participants, who are among the early preteens, were targeted. The initial sample size of PSKC was 2,150 in 2008, with the survey consisting of responses from guardians, children, parents, and school-teachers. Until the 11th data of 2018 used in this study, 1,097 participants, who were either guardians, children, parents, or school-teachers, responded to the survey. The variables used in this study were answered by children, parents, and teachers, and 342 participants responded to all the necessary variables in this study. Therefore, 342 preteens were selected as final analysis participants in this study. Considering a previous study that showed that when the number of *n* is 200 or more in panel data, it appears almost identical to the population result (22), it is considered to be a sufficient number for panel logit analysis.

### Measurements

Among the measurements used in this study, overall happiness, self-esteem, and peer attachment were analyzed using the results of children's responses to the questionnaire, and strengths and difficulties, and school adaptation were analyzed using the questionnaire answered by the child's classroom teacher observing the child. In addition, problematic smartphone use, parental supervision, and parenting behavior were used as responses from the child's primary caregiver.

**Sociodemographic and smartphone-related characteristics**. Sociodemographic characteristics included gender, residence area, household monthly income, and mother's educational level and employment status. Smartphone-related characteristics included items on whether preteens owned a smartphone and the duration of daily smartphone use.

**Problematic smartphone use**. The level of problematic smartphone usage was measured using the “K-Scale” (Internet Addiction Diagnostic Scale) (23). Among the K scale contents, the term “Internet” has been modified to “smartphone, media.” Answered by the participants' mothers, this instrument consists of 15 items: 5 items on factor 1 (daily life disorder), 4 items on factor 3 (withdrawal), 4 items on factor 4 (tolerance), and 2 items for an unclassified factor. The questionnaire items are evaluated on a four-point Likert scale from “not at all” (1 point) to “a great deal” (4 points), with higher scores indicative of a greater risk of addiction. Using the total and summation scores for sub-domains, participants were classified as general, potential-risk, and high-risk users.

General users had a total score of <27 points, and the criteria for each factor were as follows—factor 1: < 12 points, factor 3: < 10 points, and factor 4: < 9 points. Potential-risk users had a total score of 28–29 points with a score of > 13 points for factor 1, > 11 points for factor 3, and > 10 points for factor 4. Lastly, high-risk users had a total score of > 30 points, with a score of > 14 points for factor 1, > 12 points for factor 3, and > 11 points for factor 4. In this study, the potential and high-risk groups were classified as high-risk groups, and the general group was analyzed by classifying them into the general group. Cronbach's αat wave1 is 0.83, and at wave 2 is 0.87.

### Individual factors

***Overall happiness***. Overall happiness was measured by the Child Paper Self Completion Questionnaire used in the Millennium Cohort Study (MCS) (24) and represented the happiness index of daily life such as academics, appearance, family, friends, school, and life. The questionnaire consists of six items evaluated on a four-point Likert scale from “not happy at all” (1 point) to “very happy” (4 points). High scores indicate high overall happiness. Cronbach's αat wave1 is 0.73, and at wave 2 is 0.80.

***Self-esteem***. Self-efficacy was measured using the tool of the Millennium Cohort Study (MCS) (25), which reduced the Rosenberg's self-esteem scale from 10 to 5 according to age. Questionnaire items are evaluated on a five-point Likert scale from “not happy at all” (1 point) to “very happy” (5 points). High scores indicate high self-esteem. Cronbach's αat wave 1 is 0.75, and at wave 2 is 0.81.

***Strengths and difficulties***. Strengths and difficulties were measured using the Korean version of the Goodman's Strengths and Difficulties questionnaire provided on the author's website (26). PSKC researchers conducted a Korean language feasibility test to increase respondents' understanding. Measurements were based on observations made by the classroom teacher on the child's behavior over the past 6 months. Children's strengths and difficulties are divided into prosocial behavior and total difficulties. Difficulties are classified into internalizing and externalizing problems. Internalizing problems are emotional symptoms and peer relationship problems; externalizing problems are behavioral problems and hyperactivity/inattention. The tool consists of 25 items evaluated on a four-point Likert scale from “not at all” (1 point) to “a great deal” (4 points). Cronbach's αat wave1 is 0.89, and at wave 2 is 0.87.

***School adjustment***. School adjustment was measured using Ji and Jung's school adjustment inventory (27). Data were collected from participants' teachers through an online questionnaire. The tool comprises 35 items in four sub-domains: 11 items on adjustment to school life, 11 items on adjustment to academic performance, 8 items on peer adjustment, and 5 items on teacher adjustment. Each item is evaluated on a five-point Likert scale from “strongly disagree” (1 point) to “strongly agree” (5 points). High scores indicate better school adjustment. Cronbach's αat wave1 is 0.97, and at wave 2 is 0.97.

### Peer factors

***Peer attachment***. Peer attachment was measured by extracting only the peer attachment scale from Armsden and Greenberg's Parent and Peer Attachment Scale (28). The tool comprises 9 items in 3 sub-domains: communication, trust, and alienation. Communication implies respecting ideas when talking with friends, listening to what they have to say, and talking about concerns and problems. Trust implies building trust and being able to talk to your friends when you want to confide in them. Alienation implies feeling lonely and alone, even with friends. Each item is rated on a four-point Likert scale ranging from “strongly disagree” (1 point) to “strongly agree” (4 points), with high scores indicating a greater degree of communication, trust, or alienation. Cronbach's αat wave1 is 0.69, and at wave 2 is 0.74.

### Parental factors

***Parental supervision***. Parental supervision was measured using the “parenting behavior inventory” developed by Huo (29) and modified by Kim et al. (30). The questionnaire consisted of 4 items evaluated on a five-point Likert scale from “strongly disagree” (1 point) to “strongly agree” (5 points). High scores indicated high levels of child monitoring, understood as positive parenting behavior. Cronbach's αat wave1 is 0.75, and at wave 2 is 0.82.

***Parenting behavior***. Parenting behavior was measured using a tool developed by Robinson et al. and translated by PSKC researchers (31). It comprises 62 items in 3 sub-domains: authoritative, authoritarian, and permissive parenting behaviors. Authoritative parenting behavior included warmth & Involvement, reasoning/induction, democratic participation and good natured/easy going. Authoritarian parenting behavior included verbal hostility, corporal punishment, non-reasoning, punitive strategies, and directives. Permissive parenting behavior included lack of follow through, ignoring misbehavior and self-confidence. Items are evaluated on a five-point Likert scale from “strongly disagree” (1 point) to “strongly agree” (5 points). High scores indicate high parenting behavior for each sub-domain. Cronbach's αat wave1 is 0.93, and at wave 2 is 0.93.

### Data collection

Raw data of the PSKC are publicly downloadable from the Panel Study on Korean Children website. The first author revealed affiliation and purpose of use and received approval for data use.

### Data analysis

For the characteristics of wave 1 and wave 2, descriptive statistics were applied as frequency and percentage, mean and standard deviation, and a panel logit model was used to identify factors affecting the PSU by reflecting on participant growth and development. The error term e_it_ means an error that varies according to the panel object and time. STATA/BE 17 was used for longitudinal analysis of two-year data. Data from the 10^th^ and 11^th^ years of the survey (2017 to 2018) were case merged using the sample ID. The data were then converted to long-type panel data for analysis.

The Panel Logit Equation was:


$$\begin{array}{l} y_{\text{it}}={\text{α}}_{\text{i}}+{\text{β}}_{1}X_{1\text{it}}+{\text{β}}_{2}X_{2\text{it}}+e_{\text{it}} \end{array}$$

To judge suitability of this study model, we sequentially estimated its fixed and random-effects. The Hausman test was conducted to evaluate the endogeneity problem of explanatory variables. This test of the panel logit model showed that there was no correlation between individual unobserved heterogeneity of independent variables and the null hypothesis (Ho: difference in coefficients not systematic) was not rejected at a significance level of 5% (*p* = 0.419). Thus, a random effect model was used instead of a fixed effect model. Under these circumstances, the random error is heterogeneity-specific to a cross sectional unit. Owing to this intra panel variation, the random effects model has the distinct advantage of allowing for time-invariant variables to be included among the regressors.

## Results

### Demographic and smartphone-related characteristics of wave 1 and wave 2

A total of 342 participants, with approximately 50.3% girls and 49.7% boys with even gender distribution was considered. Children's smartphone ownership increased about 13.1% in wave 2 than in wave 1, indicating that 76.6% of participants own a smartphone. In wave 1, the daily smartphone usage time was 1.16 h, which increased to 1.48 h in wave 2. The number of participants in the high-risk group in wave 2 was 149, which was 6.8% higher than in wave 1 (Table 1).

**Table 1:** Demographic and smartphone-related characteristics of wave 1 and wave 2 (*N* = 342).

**Variables**	**Wave 1**	**Wave 2**
	***n*** **(%) or M (SD)**	***n*** **(%) or M (SD)**
**Gender**		
Boy	170 (49.7)	170 (49.7)
Girl	172 (50.3)	172 (50.3)
**Place of residence**				
Big city	139 (40.7)	137 (40.1)
Small and medium-sized cities	178 (52.0)	175 (51.2)
Rural area	25 (7.3)	30 (8.7)
**Household income (dollars/month)**		
<4,000	149 (43.6)	138 (40.3)
≥ 4,000– <7,000	164 (47.9)	170 (49.7)
≥ 7,000– <9,000	15 (4.4)	24 (7.0)
≥ 9,000	14 (4.1)	10 (3.0)
Mother graduated college or high school	263 (77.0)	263 (77.0)
**Mother's working status**		
Not working	147 (43.0)	133 (38.9)
Working	195 (57.0)	209 (61.1)
**Child's smartphone ownership**		
No	125 (36.5)	80 (23.4)
Yes	217 (63.5)	262 (76.6)
Time on smartphone (hr/day)	1.16 (0.80)	1.48 (0.92)
**Problematic smartphone use**		
General group	216 (63.2)	193 (56.4)
Risk group	126 (36.8)	149 (43.6)

M, mean; SD, standard deviation.

### Differences in variables between general and high-risk users

***Individual factors***. In wave 1 and 2, both the externalizing and internalizing problems of general and high-risk groups increased. In particular, the externalizing problems of high-risk groups increased the most.

***Peer factors***. In wave 1 and 2, the high-risk group had lower trust and communication scores and higher alienation scores than the general group.

***Parental factors***. In wave 1 and 2, the parental supervision score of the high-risk group was lower than that of the general group; authoritative parenting behavior increased, and permissive parenting behavior decreased in wave 2 compared to that in Wave 1 (Table 2).

**Table 2:** Differences in variables between the general and high-risk groups (*N* = 342).

**Variables**	**Wave 1**		**Wave 2**	
	**General**	**Risk**	**General**	**Risk**
	**M (SD)**	**M (SD)**	**M (SD)**	**M (SD)**
**Individual factors**				
Overall happiness	3.38 (0.44)	3.27 (0.45)	3.40 (0.46)	3.23 (0.46)
Self-esteem	3.53 (0.40)	3.47 (0.42)	3.54 (0.46)	3.45 (0.44)
**Strengths and difficulties**				
Internalizing problems	1.27 (0.28)	1.30 (0.30)	1.63 (0.24)	1.67 (0.24)
Externalizing problems	1.29 (0.31)	1.38 (0.35)	1.84 (0.28)	1.96 (0.33)
Prosocial behavior	2.53 (0.48)	2.43 (0.50)	2.60 (0.43)	2.43 (0.48)
School adjustment	4.20 (0.71)	3.99 (0.72)	4.28 (0.65)	4.02 (0.69)
**Peer factors**				
**Peer attachment**				
Communication	3.16 (0.55)	3.02 (0.58)	3.19 (0.55)	3.01 (0.50)
Trust	3.26 (0.60)	3.14 (0.65)	3.30 (0.62)	3.13 (0.58)
Alienation	1.97 (0.62)	2.03 (0.66)	1.83 (0.54)	1.90 (0.58)
**Parental factors**				
Parental supervision	4.77 (0.35)	4.59 (0.47)	4.71 (0.40)	4.57 (0.46)
**Parenting behavior**				
Authoritative	3.94 (0.38)	3.72 (0.30)	3.95 (0.33)	3.75 (0.38)
Authoritarian	2.29 (0.43)	2.44 (0.45)	2.22 (0.44)	2.41 (0.42)
Permissive	2.40 (0.27)	2.54 (0.28)	2.29 (0.32)	2.47 (0.31)

M, mean; SD, standard deviation.

### Protective and risk factors influencing problematic smartphone use

A panel logit model analysis was conducted for a longitudinal analysis of the effects of PSU in preteens (Table 3). Externalizing problems (individual factor), peer communication (peer factor), authoritative and permissive parenting behavior, and parental supervision (parental factor) affected preteens' PSU. Risk factors influencing preteens' PSU were the child's externalizing problems (*p* = 0.015, CI: 1.25–7.87) and permissive parenting behavior (*p* = 0.003, CI: 1.89–23.56). In contrast, protective factors influencing preteens' problematic smartphone use were peer communication (*p* = 0.023, CI: 0.20–0.89), parental supervision (*p* = 0.020, CI: 0.19–0.87), and authoritative parenting behavior (*p* = 0.001, CI: 0.04–0.39).

**Table 3:** Factors influencing problematic smartphone use in preteens (*N* = 342).

**Variables**	**OR**	**SE**	***p***	**95% CI**
**Individual factors**				
Overall happiness	0.84	0.40	0.704	0.33–2.11
Self-esteem	0.82	0.40	0.686	0.32–2.11
**Strengths and difficulties**				
Internalizing problems	0.70	0.44	0.563	0.20–2.38
Externalizing problems	3.14	1.47	0.015	1.25–7.87
Prosocial behavior	1.05	0.45	0.905	0.46–2.42
School adjustment	0.71	0.21	0.236	0.40–1.25
**Peer factors**				
**Peer attachment**				
Communication	0.42	0.16	0.023	0.20–0.89
Trust	1.05	0.37	0.882	0.53–2.10
**Alienation**	0.88	0.23	0.624	0.53–1.46
**Parental factors**				
Parental supervision	0.41	0.16	0.020	0.19–0.87
Parenting behavior				
Authoritative	0.13	0.07	0.001	0.04–0.39
Authoritarian	0.89	0.42	0.801	0.35–2.24
Permissive	6.68	4.30	0.003	1.89–23.56

OR, odds ratio; SE, standard error; CI, confidence interval.

## Discussion

This study analyzed the factors affecting PSU of preteens based on the ecological model longitudinally. The results indicated that preteens' externalizing problems and permissive parenting behavior were risk factors for preteens' PSU. In contrast, peer communication, authoritative parenting behavior, and parental supervision were protective factors against PSU. These results indicate that the characteristics of children, parenting and supervision, and peer relationships have complex effects on preteens' PSU. The prevalence of PSU among preteens in the 11th PSKC was 43.6%, which is much higher than that of elementary, middle, and high school students at 28% (10), which means that preteens should be carefully considered. In addition, the PSU ratio of the participants of this study was higher than that of 23.3% of adolescents in Europe, Asia and the United States (32). This result is considered to be related to the high global smartphone penetration rate in Korea.

Among the individual factors, externalizing problems such as hyperactivity and inattention increased the risk of PSU in preteens, whereas internalizing problems did not appear to affect the PSU of preteens. These results contradict previous studies on PSU influencing factors on adolescents (33, 34). According to a meta-analysis of the relationship between PSU and internalizing and externalizing problems in children and adolescents by age group, the internalizing problem showed a large effect size in older adolescents, and the externalizing problem showed a large effect in younger children (35). Also, looking at the longitudinal study of adolescent internalizing and externalizing problems, both problems in adolescents affect PSU at the beginning, but longitudinally, externalizing problems have a greater effect on PSU (36). Considering these results, the externalizing problem of preteens is an important factor affecting PSU, and it can be interpreted that this externalizing problem adversely affects the PSU even in later adolescents. Therefore, if there is an externalizing problem such as preteen's excessive behavior or carelessness, it will be necessary to monitor smartphone-related problems. And since these externalization problems can have a more adverse effect on PSU over time, interventions to manage externalizing problems are needed when developing programs or counseling related to PSU.

In this study, among the three components of peer attachment—trust, alienation, and communication—only peer communication was found to be a protective factor for PSU in preteens. Peer communication refers to respecting, listening to, and discussing concerns and problems with friends. This is consistent with previous studies reporting that the higher the quality of peer relationships, the lower the risk of PSU (37, 38). In contrast, another study found that the risk of PSU increases as peer attachment increases (39), which indicates that individuals excessively use smartphones to not feel isolated from their peer groups (40). On the other hand, in this study, peer trust and alienation did not appear as influencing factors on PSU, which is in contrast to the research results showing that peer trust or alienation among adolescents was a risk factor for PSU. Unlike the adolescent period, when they experience trust or alienation through peer attachment relationships, preteens appear to be different from the research results of adolescents because they are in the early stages of forming peer relationships and peer groups. Despite these conflicting results, it is important to form genuine friendships during the preteen period, when peer bonds begin to form. It is necessary to pay attention to the friendship of preteens at home and school, to examine and support them if there are any difficulties in the friendship. In addition, improving the communication skills of preteens and forming smooth friendships will help prevent PSU.

Our study found that authoritative parenting behavior, characterized as affection, participation, democratic relationship, and kindness/comfort, reduces the risk of PSU in preteens. Our findings are consistent with those of previous studies on school-age children (41–44). Authoritative parenting is a key factor in PSU because parents guide their children toward correct behavior and communicate with them about the reasons for following rules. In this study, it was found that high levels of parental supervision, monitoring children's activities, and demonstration of affection and interest in their daily activities were associated with reduced PSU in preteens. In the case of adolescents, the higher the level of parental supervision, the lower the likelihood of PSU, and the greater the degree of increased self-control (45). As such, parental supervision is identified as an important protective factor in the PSU of children and adolescents, which in turn indicates that such supervision can help children not engage in PSU by increasing their self-control. On the other hand, the permissive parenting behavior of parents increased the risk of PSU in preteens. These results are similar to those of previous studies showing that parental neglect increases the risk of PSU in children (46, 47). Based on these findings, parents should not ignore their children's misbehavior, be interested in smartphone use, communicate with their children about appropriate usage methods, and establish rules and instruct their children to follow them. In addition, it is necessary to participate in the PSU prevention program not only by the children themselves but also by the parents.

## Limitation

Although we considered the rapidly changing characteristics of growth and development using panel data and identified factors affecting PSU of preteens, our study has some limitations. First, our study participants were 9–10 years old, and included only a limited number of preteen participants. Therefore, in future studies, it is necessary to expand the study to preteen age participants. Second, this study was based on the study from an ecological point of view, but did not include community or national or institutional policies or legal influences. Since Korea has a characteristic that the penetration rate and use of the Internet and smartphones is higher than that of other countries, it is thought that it will be difficult to compare these policy influences with other countries. Therefore, in future studies, it is necessary to compare how each country's internet and smartphone-related laws affect PSU.

## Conclusion

In this study, we identified factors affecting problematic smartphone use in preteens, whose smartphone dependence and risk of problematic use have increased. It was found that the factors affecting the PSU of preteens in the transition period between childhood and adolescence include characteristics of childhood such as externalization problems and characteristics of adolescents such as peer attachment. When externalizing problems such as hyperactivity or inattention of preteens appear, monitoring for smartphone-related problems is required. Parents and school teachers should guide their children to form good peer relationships. Parents' correct guidance on their children's smartphone use should pay attention to what and how often their children use their smartphones rather than forcing them to restrict or disallowing them to use them. In addition, parents should teach their children to manage problem behaviors on their own through sufficient communication with their children. In order to diagnose and control preteens, who are vulnerable to PSUs, close observation and efforts are needed at school and at home.

## Data availability statement

The data used in this study is available to individual researchers or institutions upon approval of the Korea Institute of Child Care and Education (KICCE, https://kicce.re.kr).

## Ethics statement

Ethical approval was not provided for this study on human participants because this study is a secondary analysis study using Panel Study on Korean Children (PSKC), and the data is disclosed without personally identifiable information and is exempt from IRB deliberation. Written informed consent to participate in this study was provided by the participants' legal guardian/next of kin.

## Author contributions

JY, HS, and GH presented the idea and developed the theory. JY and HS improved the theory and supervised the study. GH conducted the initial analysis. JY conducted the further analysis and drafted the first version of the manuscript. All authors reviewed and approved the definitive version of the manuscript. All authors contributed to the article and approved the submitted version.

## Funding

This work was supported by a 2-Year Research Grant of Pusan National University.

## Conflict of interest

The authors declare that the research was conducted in the absence of any commercial or financial relationships that could be construed as a potential conflict of interest.

## Publisher's note

All claims expressed in this article are solely those of the authors and do not necessarily represent those of their affiliated organizations, or those of the publisher, the editors and the reviewers. Any product that may be evaluated in this article, or claim that may be made by its manufacturer, is not guaranteed or endorsed by the publisher.
